# Perfluoropolyether-incorporated polyurethane with enhanced antibacterial and anti-adhesive activities for combating catheter-induced infection†

**DOI:** 10.1039/d3ra07831k

**Published:** 2024-01-02

**Authors:** Yang Zhang, Guangbin Song, Can Hu, Zixu Liu, Huansen Liu, Yilei Wang, Liang Wang, Xuequan Feng

**Affiliations:** a School of Chemistry and Chemical Engineering, Tianjin University of Technology Tianjin China wangliang@tjut.edu.cn wang0549@e.ntu.edu.sg; b Neurosurgery Department, Tianjin First Centre Hospital Tianjin China fengxuequan@126.com

## Abstract

To avoid the undesired bacterial attachment on polyurethane-based biomedical devices, we designed a class of novel perfluoropolyether-incorporated polyurethanes (PFPU) containing different contents of perfluoropolyether (PFPE) segments. After blending with Ag nanoparticles (AgNPs), a series of bifunctional PFPU/AgNPs composites with bactericidal and anti-adhesion abilities were obtained and correspondingly made into PFPU/AgNPs films (PFPU/Ag-F) using a simple solvent-casting method. Due to its highest hydrophobicity and suitable mechanical properties, PFPU8/Ag-F containing 8 mol% of PFPE content was chosen as the optimized one for the next antibacterial assessment. The PFPU8/Ag-F can effectively deactivate over 99.9% of *Staphylococcus aureus* (*S. aureus*) and *Escherichia coli* (*E. coli*) cells at 10^6^ CFU mL^−1^ within 30 min. Furthermore, the PFPU8/AgNPs composite was used as painting material to form a protective coating for the commercial polyurethane (PU) catheter. The as-prepared PFPU8/Ag coating exhibits high resistance to bacterial adhesion in a continuous-flow artificial urine model in an 8 day exposure. Therefore, it can be expected that the proposed PFPE-containing films and coatings can effectively prevent bacterial colonization and biofilm formation on catheters or other implants, thereby reducing the risk of postoperative catheter-induced infection.

## Abstract 2

The as-synthesized PFPU polymers can facilely form films or coatings on the polyurethane substrate by simple solvent-casting or dip-coating method. The PFPU-modified surface can effectively prevent bacterial attachment over an 8 day exposure time.

## Graphical abstract

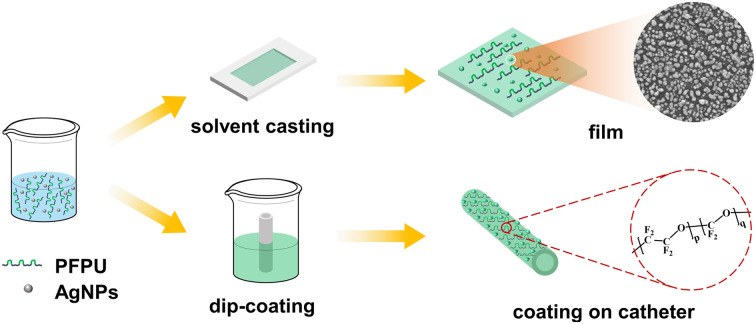

## 1. Introduction

Catheters, as common implantable medical devices, are often used for emergency or routine medical care in hospitals.^1–4^ A Foley catheter, in particular, is typically utilized to treat post-surgical symptoms related to the prostate or genital system, such as dysuria, urinary incontinence, and urinary retention.^5–8^ Its primary function is to facilitate urine excretion when the body is experiencing bladder pressure or kidney failure.^9–11^ However, during the clinical application of catheters, many patients are particularly susceptible to urinary tract infections caused by the catheter.^12–15^ Generally speaking, bacteria or other microorganisms tend to colonize and multiply on the surface of the vessel, ultimately forming a biofilm, rather than individually proliferate in the medium.^16^ The formation of biofilms increases bacterial resistance, leading to further infection and ultimately catheter obstruction in patients undergoing long-term catheterization.^17,18^ Furthermore, many complications, such as bacterial urine infection, prostatitis, septicemia, and shock, also arise as a consequence of the biofilm.^19–21^

Current strategies for preventing catheter-induced infections mainly focus on the development of novel antibacterial interfaces with bactericidal, anti-fouling, or bifunctional-combined properties.^22–26^ Numerous antibacterial substances including quaternary ammonium salts, antibiotics, peptides, polylysines, chitosan and carbon dots have been utilized to enhance the bactericidal capacity of the target surface.^27–31^ For instance, several quaternary ammonium compounds with a thiol group were covalently coupled to the PU surface using an efficient thiol–ene “click” reaction. Antibacterial assays revealed that the PU surface modified by compound Q8-SH possessed the highest ability to kill adherent bacteria. It could rapidly kill over 75% of *S. aureus* within 5 min and 90% of *E. coli* within 10 min.^32^ In another work, by using surface ring-opening polymerization, antibacterial poly-l-lysine (PLL) was *in situ* grown on the surface of the catheter to form a brush-like polymeric layer. The resulting PLL brush-covered catheter retained its bactericidal abilities for 65 days in simulated body fluid.^33^ Kara *et al.* introduced chitosan and heparin into hexamethylene diisocyanate-based polyurethanes to impart antibacterial properties to the PU for medical devices. This material showed substantial antibacterial and anti-adhesive efficacy against both Gram-positive and Gram-negative bacteria.^34^ Das *et al.* immobilized heteroatom-doped carbon dot in biopolymer composites for functional textiles. The carbon dots exhibited antioxidant and antimicrobial properties, effectively inhibiting bacterial growth and offering a potential solution for healthcare-related contamination prevention.^35^ However, to date, only a few antibacterial agents, such as certain antibiotics and silver, have entered clinical practice to inhibit catheter-induced infections.^36,37^ On the other hand, the anti-fouling modification for the surface of device to prevent bacterial cell attachment is also important. To realize this purpose, hydrophobic, zwitterionic, and amphiphilic polymers were applied to specific surfaces, as well as employing bioinspired techniques to prevent bacterial colonization.^38^ Wherein low surface energy surfaces with micro/nanostructures and chemical modifications, which have high liquid repulse ability, can prevent microbial adhesion and delay the formation of biofilms. These surfaces typically exhibit superhydrophobic or superoleophobic properties or repellency to liquids. Zhao *et al.* designed a hydrophobic and electroless surface covered by silver–polytetrafluoroethylene coating and reported that the surface with approximately 24.5 mN m^−1^ surface energy exhibited the highest performance towards minimizing bacterial adhesion.^39,40^ However, some recent works reported that the optimal surface tension of 24.5 mN m^−1^ may not be suitable for all situations.^41^ For example, superhydrophobic coatings were established on silicon substrates using a dip-coating method with a tetrahydrofuran solution consisting of polydimethylsiloxane, perfluorosilane-coated hydrophobic zinc oxide, and copper nanoparticles as hydrophobic painting materials. The coatings can significantly reduce bacterial adhesion and biofilm formation of *E. coli* compared to control groups.^42^ Such superhydrophobic coatings constructed by micro/nanostructures are usually unstable. In the complex *in vivo* environment, with the detachment of nanoparticles, the irreversible adsorption of proteins will happen, which will further facilitate bacterial colonization through specific protein–bacterial interactions on the targeting surface.^43,44^

To solve these problems, we designed a facile, efficient, and versatile strategy for developing bifunctional polyurethane films and coatings with enhanced bactericidal and anti-adhesive activities. First, five PFPU samples were obtained and respectively designated PFPU0, PFPU4, PFPU8, PFPU16, and PFPU20 according to their PFPE contents. Second, these polymers were blended with AgNPs and prepared into five film samples (PFPU0/Ag-F, PFPU4/Ag-F, PFPU8/Ag-F, PFPU16/Ag-F, and PFPU20/Ag-F). By screening the hydrophobicity and mechanical properties, the PFPU8/Ag-F was chosen as the optimized one for the next antibacterial tests. Third, the anti-adhesion assessments were performed on PFPU8/Ag coating. In the above design, the AgNPs provide bactericidal properties while the PFPU8 acts as not only an antifouling layer but also an adhesive to bond the PFPU8/Ag coating on the surface of the PU catheter. Compared to other surface modification strategies,^45–47^ our polymer-based materials mainly have three advantages. The first one is the high stability of the coating which is given by the uniform coating layer from the single PFPU8 polymer; the second one is the self-lubricating property which is endowed by the PFPE segment; the last one is the long-term anti-adhesive property.

## 2. Experimental

### 2.1 Materials

1,4-Butanediol (BDO), methylene-bis(4-cyclohexylisocyanate) (HMDI), stannous octoate, and CDCl_3_ were purchased from Energy Chemical Co. Ltd (Shanghai, China). Poly(ethylene glycol)-*block*-poly(propylene glycol)-*block*-poly(ethylene glycol) (Pluronic L-81, average *M*_n_ ∼ 2800) and ethidium bromide (EB) were purchased from Sigma-Aldrich (St. Louis, MO, USA). Poly(perfluoroethoxy)difluoroethyl PEG ether (E10H, *M*_n_ ∼ 1700) and acridine orange (AO) were purchased from Alfa Aesar Chemical Co. Ltd (Shanghai, China). Tryptone, yeast extract powder, agar powder, and glucose were purchased from Beijing Oboxing Biotechnology Co., Ltd (Beijing China). Phosphate buffer saline (PBS) was purchased from Beijing Solarbio Science & Technology Co., Ltd (Beijing China). Other solvents were all purchased from Tianjin Damao Chemical Reagent Factory (Tianjin, China). Oil-soluble AgNPs (50 nm) was purchased from Nanjing XFNANO Materials Tech Co., Ltd (Nanjing, Jiangsu, China). All commercially available reagents and solvents were used directly without further purification.

### 2.2 Synthesis of PFPU

By verifying the E10H feeding content from 0 to 4, 8, 16, and 20 mol%, five PFPU samples were prepared. The following is provided as an example procedure for PFPU8. 0.92 g (9.12 mmol) of BDO, 3.82 g (1.37 mmol) of L-81, and 1.55 g (0.91 mmol) of E10H were preheated to 100 °C in a round-bottomed flask with electromagnetic stirring. Then, 2.99 g (11.40 mmol) of HMDI was added, immediately followed by a drop of stannous octoate catalyst. The mixture was stirred for 2–3 min until it became too viscous to stir. The polymerization system was further oven-cured at 100 °C for 24 h to give the crude product as a white elastomer. The obtained PFPU8 was further purified by the dissolving-precipitation method using THF as a good solvent and ethyl ether as a poor solvent.

### 2.3 Preparation of PFPU/Ag-F

The solvent casting method was used to prepare the PFPU/Ag-F. The procedure for PFPU8/Ag-F is taken for example. Briefly, 0.50 g of PFPU8 and 2.50 mg of oil-soluble AgNPs were dissolved in 5 mL of THF to form a yellow solution. The solution was then cast in a Teflon Petri dish (40 mm × 60 mm) and dried at room temperature for 24 h. After most of the solvent evaporated, the film was placed under vacuum at 60 °C for 48 h. The above method was also used to prepare PFPU-F without AgNPs addition.

### 2.4 Antibacterial activity of PFPU8/Ag-F

Antibacterial activities of PFPU8/Ag-F were determined by quantifying the inactive rate of bacteria held in intimate contact towards the film sample for 10, 20, 30, and 60 min at 37 °C towards the film sample according to ISO 22196 standard.^48^*E. coli* and *S. aureus* were used as the bacterial models for Gram-negative and Gram-positive bacteria. The PFPU8-F without an antibacterial agent was used as a control group while the PFPU8/Ag-F was the testing film. The experimental details are as follows. The film samples (20 mm × 20 mm) sterilized by ultraviolet (UV) treatment were placed into sterile Petri dishes. 20 μL of bacterial solution (10^6^ CFU mL^−1^) was pipetted onto the film samples. Then the inoculated film samples were covered with a piece of UV-sterilized plain polyethylene (PE) film (10 mm × 10 mm). Petri dishes containing the inoculated film samples were incubated at 37 °C under a relative humidity of above 90% for the scheduled period. Microorganisms were retrieved from film samples by sonication bath and dispersed in PBS solution and then inoculated onto tryptic soy agar plates. After incubation at 37 °C for 16 h, colonies grown on plates were counted and the loss of viability was calculated and presented as the percentage of dead bacteria compared with the control group. Results are reported as averages with mean ± standard deviation (SD) (*n* = 5) for each sample.

### 2.5 Bacterial morphology on the PFPU8/Ag-F

The bacteria on PFPU8-F and PFPU8/Ag-F were observed by scanning electron microscope (SEM). Samples for SEM were prepared using the method described as follows. After incubating with bacterial cells for 30 min, the film samples were gently washed with PBS and immersed into 2.5 wt% glutaraldehyde solution to fix the bacteria cells attached to them. The films were then gradually dehydrated with a series of ethanol/water mixed solutions with the ethanol concentrations ranging from 10, 30, 50, 70, and 90 vol% to pure ethanol, and then freeze-dried under vacuum. The as-prepared film samples were examined by the SEM under the electron acceleration voltage of 10.00 kV.

### 2.6 Integrity of bacterial cell membrane

The cell membrane damage after incubation with PFPU8-F and PFPU8/Ag-F for 30 min was evaluated by AO/EB double staining assay. Briefly, the bacterial cells were detached from the film by sonication bath and re-dispersed in 1 mL of PBS and then collected by centrifugation at 3000 rpm for 5 min. The collected pellets were washed and re-dispersed in 1 mL of PBS and then pipetted 20 μL of the bacterial suspension on a glass slide and stained with 1 μL of AO/EB (6 mg mL^−1^ of AO and 10 mg mL^−1^ of EB) for 15 min in dark.^49^ A confocal laser microscope was then employed to observe the above samples. Bacteria cells with intact membranes were stained green, whereas cells with compromised membranes that were considered to be dead or dying were stained red.

### 2.7 Preparation of PFPU8/Ag coating on the catheter

First, 0.50 g PFPU8 and 0.25 mg oil-dispersed AgNPs were dissolved together in 10 mL THF to form the painting solution. Second, the commercial PU catheter was cut into 5 cm long pieces and each piece was immersed in the painting solution for 10 seconds, and then lifted slowly. After natural drying in the air, the catheter samples with PFPU8/Ag coatings were further dried in a vacuum oven at 40 °C for 12 h. In addition, the PFPU0 and PFPU8 coatings were also prepared as the control groups according to the same procedure as above.

### 2.8 Anti-adhesion testing of PFPU8/Ag coating

The anti-adhesion assay was performed in a continuous-flow artificial urea model. *E. coli* and *S. aureus* were also used as the bacterial models. Briefly, the 5 cm long catheter samples with different coatings prepared in Section 2.7 were connected with a silicone tube. A peristaltic pump was used to pump the artificial urine containing 10^6^ CFU mL^−1^ bacterial cells passing through the lumen of the catheter sample at a constant flow rate of 5.8 mL min^−1^. To better simulate the *in vivo* physiological environment, once the artificial urine was flowing through the catheter sample, it was disposed and the bacterial-contaminated artificial urine was replaced daily with freshly made one. The number of adhered bacterial cells on the inner wall of the catheter sample was examined by the plate counting method and presented in logarithmic form (log CFU cm^−2^). Data was collected as the average number of five parallel experiments for each sample and was presented as mean ± SD.

### 2.9 Characterizations

^1^H NMR spectra were obtained using a Bruker 400 MHz NMR spectrometer operated at 303 K. All chemical shifts were reported in ppm (*δ*) and referenced to the chemical shifts of residual solvent resonances (CDCl_3_: *δ* = 7.26 ppm). Gel permeation chromatography data (GPC) were obtained by gel permeation chromatograph (Malvern Viscotek HT). The mobile phase is THF, and the test temperature is 40 °C. The molecular weight was determined according to the standard sample of polystyrene. The surface morphologies of the film samples were characterized using a SEM measurement (MERLIN Compact, ZEISS). The surface chemical info of the PFPU-F was characterized by X-ray photoelectron spectroscopy (XPS) (ESCALAB250Xi, THERMO SCIENTIFIC). The surface wettability was characterized by measuring the water contact angle using a contact angle measurement instrument (SZ-CAMA1, Sunzern). Bacterial staining was observed and imaged using a confocal laser scanning microscope (A1, Nikon Instruments) under a 60× objective lens.

## 3. Results and discussion

### 3.1 Synthesis of PFPU

**Scheme 1:**
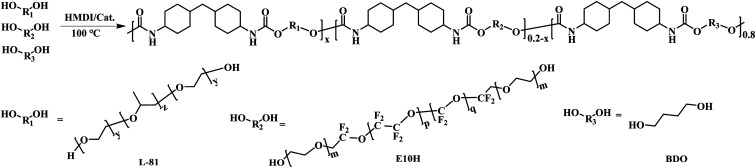
Synthetic route of PFPU polymers.

As illustrated in Scheme 1, PFPU was synthesized using a traditional polycondensation reaction between HDMI and three diols including L81, E10H, and BDO. Among them, L81 and E10H constitute the soft segment of PFPU while BDO acted as the hard segment. GPC tests were used to analyze the molecular weight and distribution of PFPU polymers. By tuning the feeding content of E10H from 0 to 4, 8, 16, and 20.0 wt%, five PFPE samples were obtained. Their synthetic information and molecular weight are listed in Table 1. As shown in Table 1, upon increasing E10H content in the feed, the yield and the molecular weight decrease. It could be seen that when E10H feeding content increases from 0% to 20%, the yield and *M*_w_ respectively decrease from 98.2% to 91.2% and from 42 140 to 14 693. Due to its low solubility in the reaction system, E10H exhibits relatively low reactivity. This might be the reason for the decrease in the yield and molecular weight.

**Table 1:** Molecular weight of PFPU polymers with the E10H feeding variation

Sample	E10H feeding content (mol%)	Yield (%)	Molecular weight^a^		
			*M*_n_	*M*_w_	PDI
PFPU0	0	98.2	25 421	42 140	1.66
PFPU4	4	97.5	24 676	39 397	1.60
PFPU8	8	96.3	19 591	32 247	1.65
PFPU16	16	96.5	9879	16 300	1.65
PFPU20	20	91.2	8771	14 693	1.68

a Determined by GPC.

**Fig. 1:**
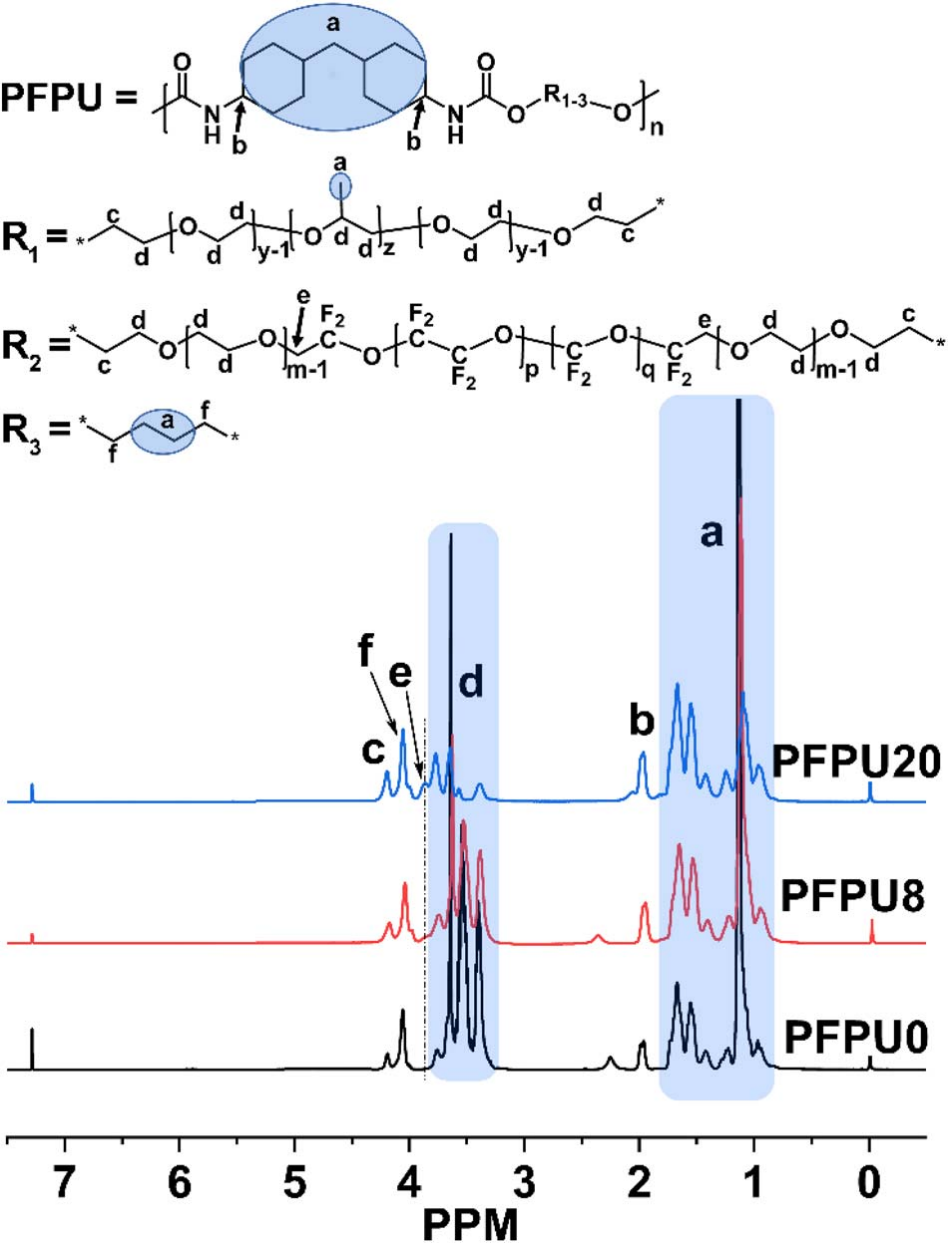
^1^H-NMR spectra of PFPU0, PFPU8 and PFPU20.

^1^H NMR was used to characterize the proportion and structure of PFPU polymers. Fig. 1 displays the ^1^H NMR spectra of the PFPU0, PFPU8, and PFPU20. Due to the similar repeating units in the L81 and E10H segments (–CH_2_–CH_2_–O–), some hydrogen signals overlapped and were hard to define separately. In comparison with the spectra of PFPU0 and PFPU20 (Fig. 1), the analysis of the ^1^H NMR spectrum of PFPU8 is illustrated as follows. The peaks (a) in the range from 0.80 to 1.82 ppm are contributed by the CH_2_ and CH groups in the BDO, HMDI, and L81 units which are not connected to a heteroatom (Fig. 1). The peak (b) at 1.95 ppm is assigned to the hydrogen signal of the C**H** groups in HDMI units which are connected to the nitrogen atom of the urethane groups. Two peaks (f and c) at 4.03 ppm and 4.17 ppm are contributed by the C**H**_2_ groups in three types of diol units (BDO, L81, and E10H) which are connected to the oxygen atom of the urethane groups. The appearance of above signals confirms the formation of urethane groups in the polymerization. The multiple peaks (d) from 3.06 to 3.81 ppm are all from the –C**H**_2_–C**H**_2_–O– repeating units in both L81 and E10H segments. Notably, the weak peak (e) which appears only in the spectra of the E10H-containing PFPUs (PFPU8 and PFPU20) is attributed to the hydrogen signal of –C**H**_2_–CF_2_– groups, indicating the successful incorporation of E10H segments in the PFPU mainchain.

### 3.2 Surface and mechanical properties of PFPU films

All of the obtained PFPU polymers were prepared into films by a simple solvent-casting method. Five PFPU film samples were obtained and were respectively named as PFPU0-F, PFPU4-F, PFPU8-F, PFPU16-F, and PFPU20-F. After the addition of AgNPs, another five PFPU/Ag film samples were prepared. They are correspondingly named as PFPU0/Ag-F, PFPU4/Ag-F, PFPU8/Ag-F, PFPU16/Ag-F, and PFPU20/Ag-F.

**Fig. 2:**
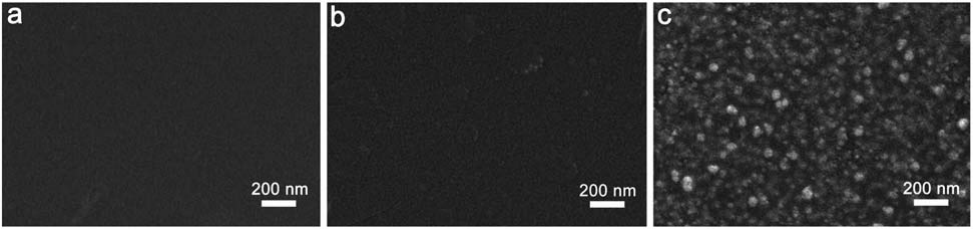
SEM images of PFPU0-F (a), PFPU8-F (b), and PFPU8/Ag-F (c).

To reveal the effect of surface toughness on the hydrophobicity of the films, the PFPU0-F, PFPU8-F, and PFPU8/Ag-F were visualized and compared using SEM (Fig. 2). As demonstrated in Fig. 2a, the PFPU0-F without PFPE segments exhibits a smooth surface. Compared to the PFPU0, the PFPU8-F has a relatively rough surface (Fig. 2b). Because of their high repellence, the PFPE segments in PFPU8 tend to aggregate at the interface between the film and air and form such surface morphology as shown in Fig. 2b. After AgNPs addition, significant nanoscale morphologies were observed in the SEM image of PFPU8/Ag-F (Fig. 2c). The AgNPs with around 50 nm diameter are randomly distributed on the surface or embedded in the film. This nanoscale roughness is helpful to our research because it can increase the surface hydrophobicity of PFPU8/Ag-F and subsequently reduce the bacterial attachment.

**Fig. 3:**
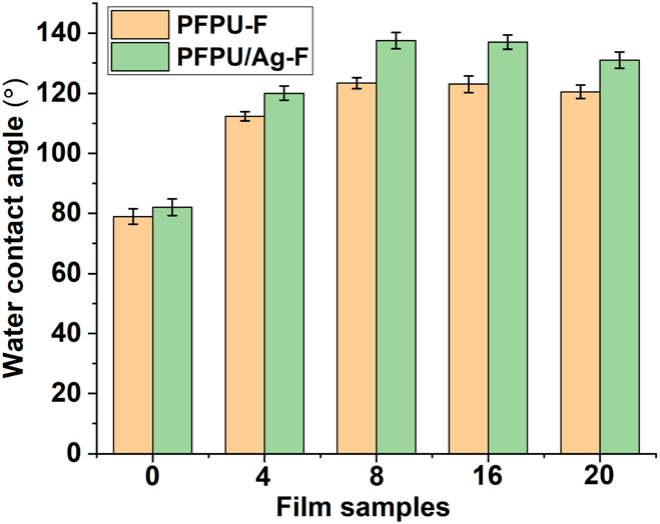
Water contact angles of PFPU-F and PFPU/Ag-F with E10H feeding variation. The results with error bars reported as mean ± SD (*n* = 5) for each sample.

Surface hydrophobicity of the films were evaluated by water contact angle measurement. Fig. 3 shows the water contact angle data of these films. As for the film series without AgNPs (Fig. 3), the addition of E10H dramatically elevated the hydrophobicity of the films so that the addition of 4 mol% E10H resulted in the value of the water contact angle increasing from 78.9° to 112.4°. During the film-forming process, the fluorine segments can spontaneously generate orientation, forming low surface energy surfaces. The low compatibility of PFPE segments drove them to gradually aggregate at the film surface. Therefore, the addition of a small amount of E10H can greatly reduce the surface energy, resulting in a significant increase in the water contact angle. By continuing to increase the feeding content of E10H from 4 mol% to 20 mol%, all of the three film samples (PFPU8-F, PFPU16-F, and PFPU20-F) show higher hydrophobicity than that of PFPU4-F (Fig. 3). Interestingly, the highest water contact angle (123.3°) was found in PFPU8-F but not in PFPU20-F. Theoretically, lower molecular weight leads to higher content of end groups in the polymer. As for the PFPU polymers, the end groups are hydrophilic hydroxyl or amino, which will increase the hydrophilicity of the film. So, the lowest molecular weight of PFPU20 (*M*_n_ = 8771) might be the reason for the reduction of water contact angle in PFPU20-F. After the addition of AgNPs (Fig. 3), the water contact angles of PFPU/Ag-F are all higher than those of the corresponding PFPU-F films. This might be caused by the surface nano-morphology of the PFPU/Ag-F.^50^ Notably, the highest water contact angle is 137.5° among these PFPU/Ag-F samples, which belongs to PFPU8/Ag-F.

**Fig. 4:**
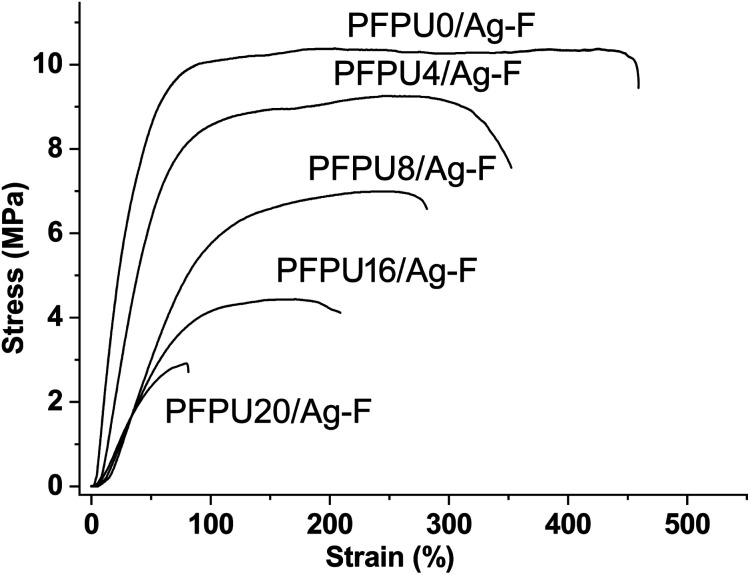
Stress and strain curves of PFPU/Ag-F with E10H feeding variation.

To evaluate the effect of E10H content on the mechanical properties of PFPU/Ag-F, strain–stress curves were measured and shown in Fig. 4. The tensile strength of PFPU/Ag-F samples decreased from 10.5 to 3 MPa when the E10H feeding content increased from 0 to 20 mol%, which is mainly due to the decrease in molecular weight. At the same time, the elongation at break is also reduced significantly from 450% to 80% as the E10H feeding content increases from 0 to 20 mol%, suggesting that E10H has less contributions to the flexibility of the PFPU polymer than the L81 monomer. Because the PFPU20/Ag-F shows high rigidity and fragility, no further research will be conducted on this sample. Due to its highest water contact angle, appropriate molecular weight, and moderate tensile strength and elongation rate, PFPU8/Ag-F was selected as the optimized one for the subsequent antibacterial and anti-adhesive studies.

**Fig. 5:**
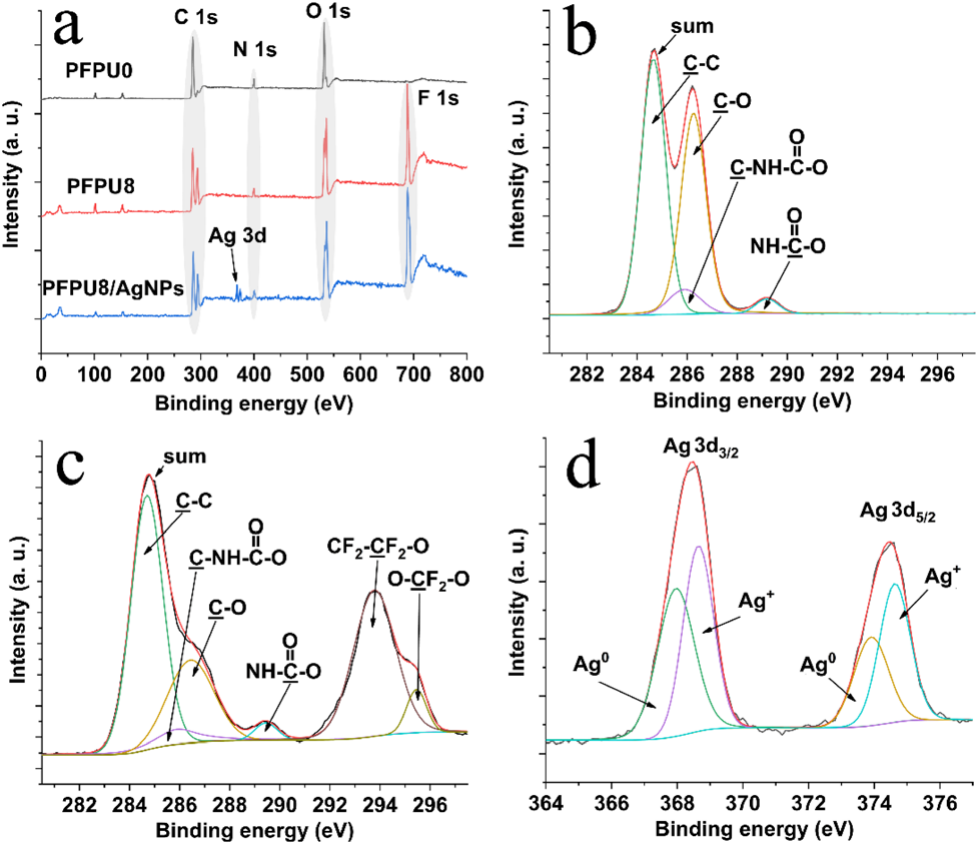
XPS wide scan spectra of PFPU0-F, PFPU8-F, and PFPU8/Ag-F (a), C 1s spectrum of PFPU0-F (b), C 1s spectrum of PFPU8-F (c), and Ag 3d spectrum of PFPU8/Ag-F (d).

XPS was employed to examine the surface chemical composition of PFPU0-F, PFPU8-F, and PFPU8/Ag-F (Fig. 5). The wide scan spectrum of PFPU0-F (Fig. 5a) shows regular C 1s, O 1s, and N 1s peaks which emerge at around 285, 400, and 532 eV, respectively. Compared with PFPU0-F, a strong peak appears at 688 eV in the spectrum of PFPU8-F, indicating that PFPU8-F has abundant fluorine elements on its surface. It can be observed from the spectrum of PFPU8-F in Fig. 5a that the C 1s peak is divided into two peaks respectively at 285 and 293 eV. The new peak at 293 eV is contributed by the –C̲F_2_– units from the PFPE segments. These results strongly suggest that the PFPE segments are highly enriched on the surface of PFPU8-F, consistent with the SEM and water contact angle results. The XPS wide scan spectrum of PFPU8/Ag-F (Fig. 5a) shows similar binding energy and intensity in the positions of C 1s, O 1s, and N 1s. The difference is that a double peak of Ag 3d emerges at around 369 eV, which originates from the AgNPs addition. To further identify the carbon-related groups, the high-resolution C 1s spectrum of PFPU0-F (Fig. 5b) was curve fitted to four peaks with the binding energy at 284.6, 285.9, 286.3, and 289.2 eV, which can be assigned to the carbons in C–C̲, C̲–O, C̲–NH–C=O, and C–NH–C̲=O groups, respectively, consisting with the polyurethane structure. Besides, the C 1s spectrum of PFPU8-F can be divided and fitted to six peaks. Among them, four peaks at 284.6, 285.9, 286.3, and 289.4 eV also come from the four types of carbon as illustrated in Fig. 5b, while two new peaks at 293.8 and 295.4 eV can be respectively assigned to the CF_2_–C̲F_2_–O and O–C̲F_2_–O groups, which is consisting with the PFPE component. Furthermore, the peak deconvolution of Ag 3d (Fig. 5d) reveals the binding energies from both metallic (Ag^0^) and oxidized (Ag^+^) species.^51,52^ The latter is probably caused by the surface partial oxidation upon exposure to air.

### 3.3 Antibacterial properties of PFPU8/Ag-F

**Fig. 6:**
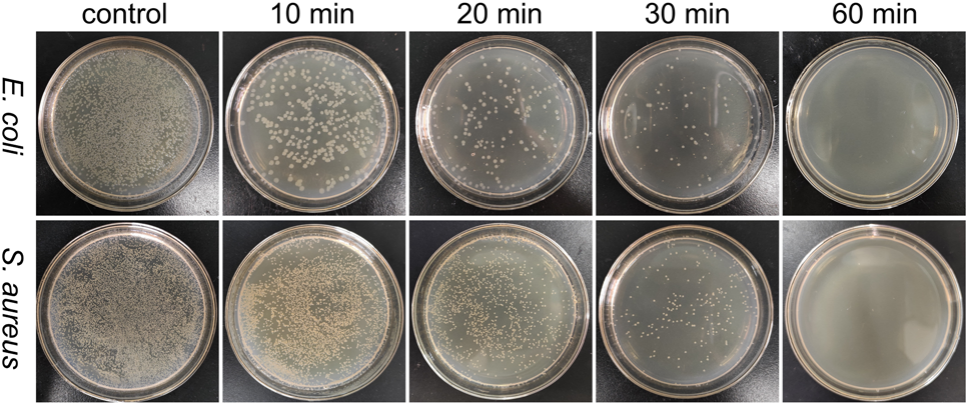
Agar plate images of *S. aureus* and *E. coli* after incubating with PFPU/Ag-F for 10, 20, 30, and 60 min.

**Fig. 7:**
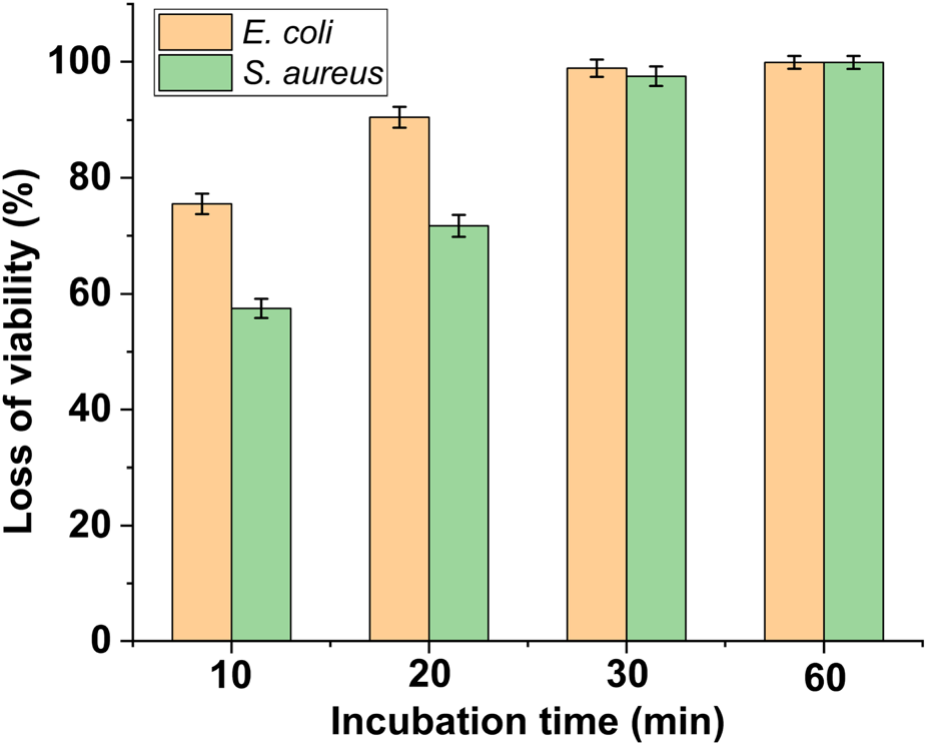
Loss of viability of *S. aureus* and *E. coli* after incubating with PFPU/Ag-F for different durations. The results with error bars reported as mean ± SD (*n* = 5) for each sample.

To evaluate the antibacterial activity of PFPU8/Ag-F, *E. coli* and *S. aureus* cells at the density of 10^6^ CFU mL^−1^ were inoculated on its surface with the incubation time varied from 10, 20, 30, to 60 min. As shown in Fig. 6 and 7, the loss of viabilities related to both types of bacterial cells show an upward trend with the time extension, indicating that the PFPU8/Ag-F can effectively inactivate the bacterial cells over a certain period. When the incubation time is longer than 30 min, the PFPU8/Ag-F can kill most of the contact bacterial cells and the loss of viabilities are all higher than 95% regardless of the bacterial species. It also can be observed that the loss of viabilities related to *E. coli* are all higher than those related to *S. aureus* within 30 min incubation. Many studies have shown that AgNPs have a more significant bactericidal effect on Gram-negative bacteria compared to Gram-positive bacteria.^53^ It is reported that the difference is caused by the distinct structural compositions of the cell walls of the two bacterial species, resulting in variable sensitivity to AgNPs.^54^ The cell walls of Gram-positive bacteria have multiple layers of peptidoglycan (30 nm thick peptidoglycan layers), while the cell walls of Gram-negative bacteria have only a single layer of 2–3 nm peptide-based layers, which are covered by an outer membrane.^55^ Therefore, the thick cell walls of Gram-negative bacteria may receive more Ag^+^ than that of Gram-positive bacteria, which leads to better sensitivity to the antibacterial activity of silver antimicrobial agents.

**Fig. 8:**
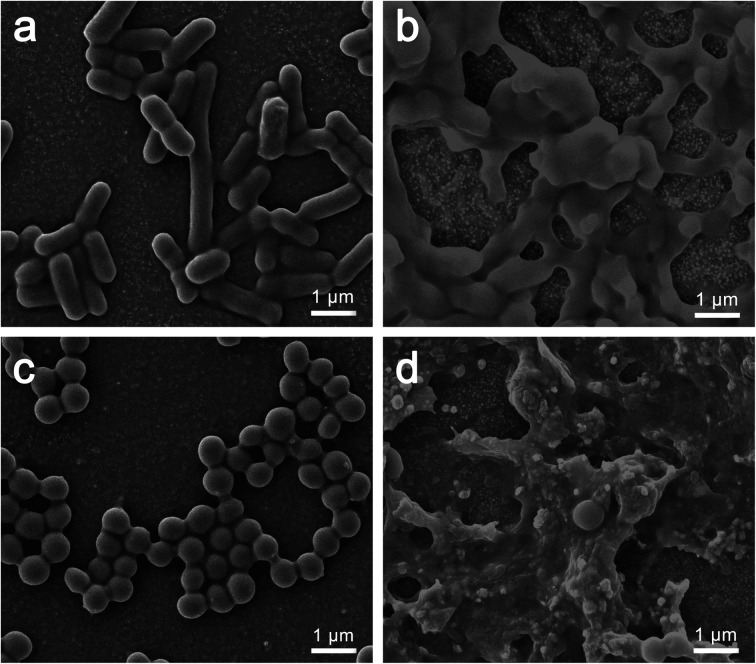
Morphologies of bacterial cells after 30 min incubation: *E. coli* cells on PFPU8-F (a) and PFPU8/Ag-F (b); *S. aureus* cells on PFPU8-F (c) and PFPU8/Ag-F (d).

SEM was used to observe morphological changes of *E. coli* and *S. aureus* cells upon interacting with PFPU8-F and PFPU8/Ag-F for 30 min. Fig. 8a demonstrates that *E. coli* cells present a smooth surface and a rod-like shape on PFPU8-F, indicating that only PFPU film cannot induce significant damage to bacterial cells. In contrast, upon incubating with PFPU8/Ag-F, most *E. coli* cells lose their inherent morphology and become collapsed and glued together into a lump (Fig. 8b). Under the same conditions, *S. aureus* cells on PFPU8-F are regular round spheres (Fig. 8c), however, most of them on PFPU8/Ag-F are unable to distinguish (Fig. 8d). Overall, the SEM results further confirm the bactericidal ability of the PFPU8/Ag-F and also reveal that bacterial death is probably induced by the cell membrane rupture.

**Fig. 9:**
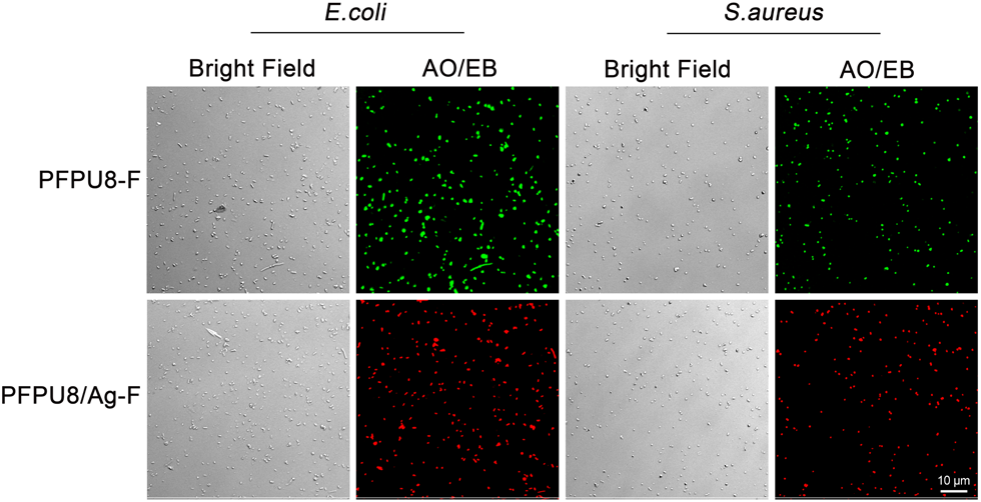
Cell wall damage assessed by AO/EB staining. *E. coli* and *S. aureus* cells after incubating with PFPU8-F and PFPU8/Ag-F for 30 min.

Cell membrane integrity of *E. coli* and *S. aureus* cells after incubating with PFPU8-F and PFPU8/Ag-F for 30 min was further investigated using fluorescent dyes (AO/EB). Live bacterial cells with inherent cell walls can be stained by AO and emit green luminescence. EB can only penetrate cells when their membranes are damaged.^56^ As shown in Fig. 9, both of the two types of bacterial cells incubated with PFPU8-F show bright green fluorescence, indicating that the bacterial cell membrane is intact and the cells are live. In contrast, these bacterial cells incubated with PFPU8/Ag-F look like many red dots under UV excitation, suggesting disruption of the cell membrane. AgNPs have demonstrated effective antimicrobial activity against microorganisms. However, the antibacterial mechanisms of AgNPs remain elusive to researchers. Some mechanisms attribute the antibacterial activity to membrane damage induced by AgNPs, while others propose the release of silver ions from the surface of nanoparticles.^57,58^ Recently, it was reported that the bactericidal activity of AgNPs is from a synergistic effect between the contact of AgNPs and the release of Ag^+^. This mechanism suggests that direct cell-nanoparticle contact facilitates the release of Ag^+^ from AgNPs, thereby enhancing the amount of cellular uptake of the released Ag^+^.^59^ Nevertheless, from the results of SEM and bacterial stain assay, the reason for the bacterial death in our research seems to be induced by cell wall rupture.

### 3.4 Anti-adhesion ability of the PFPU8/Ag coating

**Fig. 10:**
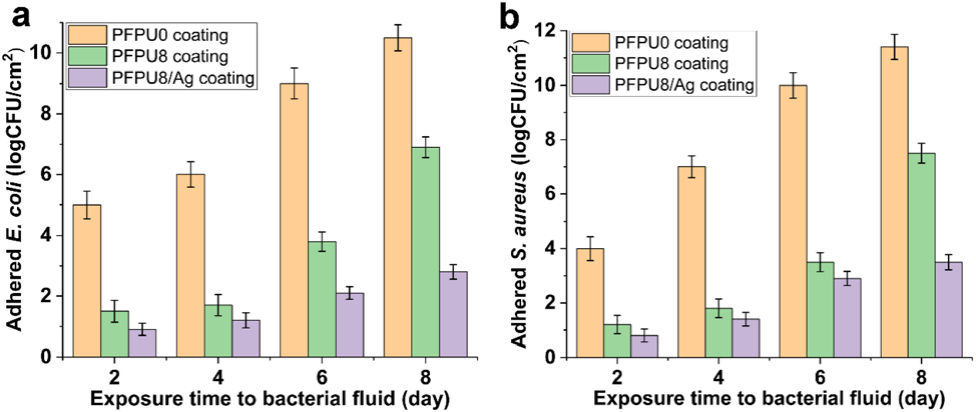
Logarithmic *E. coli* (a) and *S. aureus* (b) cell population adhered to the PFPU0, PFPU8, and PFPU8/Ag coatings after different exposure time. The results with error bars reported as mean ± SD (*n* = 5) for each sample.

The efficacy of PFPU8/Ag coating to inhibit bacterial adhesion was examined on the commercial catheter for 2, 4, 6, and 8 days in a flow of artificial urine driven by a peristaltic pump. A low shear flow condition was simulated by continuous drip-flow of artificial urine to mimic *in vivo* conditions. The PFPU0 coating demonstrates a poor antibiofilm effect against *E. coli* (Fig. 10a yellow column). In contrast, the PFPU8 coating shows a lower density of adhered bacterial cells at each point of time, compared with PFPU0 coating, indicating the PFPE segments can strongly prevent bacterial adhesion (Fig. 10a green column). As we anticipated the PFPU8/Ag coating is much more effective in protecting of the catheter from bacterial adhesion than the former two coatings (Fig. 10a purple column) due to the synergistic effect of high surface repellence and good antibacterial ability. After 8 days of exposure to the bacteria-containing artificial urine, the attached bacterial population on PFPU8/Ag coating is reduced by 7.7 log_10_ compared to that on the PFPU0 coating. Furthermore, the adhesion experiments of *S. aureus* cells on the three types of coatings (Fig. 10b) draw similar trends in the antifouling ability, where PFPU8/Ag coating had the lowest adhered-bacterial population and exhibits 7.9 log_10_ reduction compared to the PFPU0 coating after 8 days.

## 4. Conclusions

In this study, PFPE segments were successfully introduced into the polyurethane mainchain by traditional polycondensation between hydroxyl and isocyanate groups, and five PFPU polymers with PFPE feeding content ranging from 0, 4, 8, 16, and 20 mol% were obtained. Five PFPU/Ag-F films containing 0.5 wt% AgNPs were prepared from the corresponding PFPU polymers by a solvent-casting method. Among them, the PFPU8/Ag-F was chosen for antibacterial and anti-adhesion evaluation because it has the highest water contact angle of 137° and moderate tensile strength and elongation rate of 6.4 MPa and 265%. In the antibacterial test, the PFPU8/Ag-F can kill most of the attached bacterial cells with the inactivating rate being higher than 95% after 30 min incubation. As revealed by SEM and bacterial stain assay, the reason for bacterial death might be cell wall damage. Furthermore, the PFPU8/Ag coating on the commercial catheter presents 7.7 log_10_ and 7.9 log_10_ reductions in preventing *E. coli* and *S. aureus* attachment over an 8 day period in the continuous-flow artificial urine model. More importantly, compared to other reported surface-modification approaches,^42,60–62^ our proposed film or coating is accomplished by a single polymer (PFPU), which maximum guarantees the stability and durability of the anti-adhesion effect. In addition, the dip-coating method is simple and highly applicable to various existing catheters regardless of their geometry and size, making it possible to apply this technology to various implantable devices. Overall, due to its long-term antifouling, stability, and excellent antibacterial properties, the PFPU8/Ag coating has great potential for use in preventing catheter-induced infection.

## Author contributions

Yang Zhang: validation, methodology, formal analysis, writing original draft. Guangbin Song: validation, methodology, formal analysis, writing original draft. Can Hu: validation, methodology. Zixu Liu: methodology, synthesis of PFPU. Huansen Liu: characterization of mechanical properties for PFPU. Yilei Wang: writing – review& editing. Liang Wang: conceptualization, formal analysis, writing original draft, writing review & editing, funding acquisition. Xuequan Feng: resources of anti-bacterial and anti-adhesive experiment, writing review & editing.

## Conflicts of interest

There are no conflicts to declare.

## Supplementary Material

RA-014-D3RA07831K-s001
