# Supplementary material for: Perfluoropolyether-incorporated polyurethane with enhanced antibacterial and anti-adhesive activities for combating catheter-induced infection
Source: RSC Adv. 2024 Jan 2;14(1):568–76. doi: 10.1039/d3ra07831k (PMC10759042; doi:10.1039/d3ra07831k)
Supplement: RA-014-D3RA07831K-s001 [file RA-014-D3RA07831K-s001.pdf]

Electronic Supplementary Material (ESI) for RSC Advances.
This journal is © The Royal Society of Chemistry 2023

All of the NMR data were produced by MestRe-C and copied in their original form.

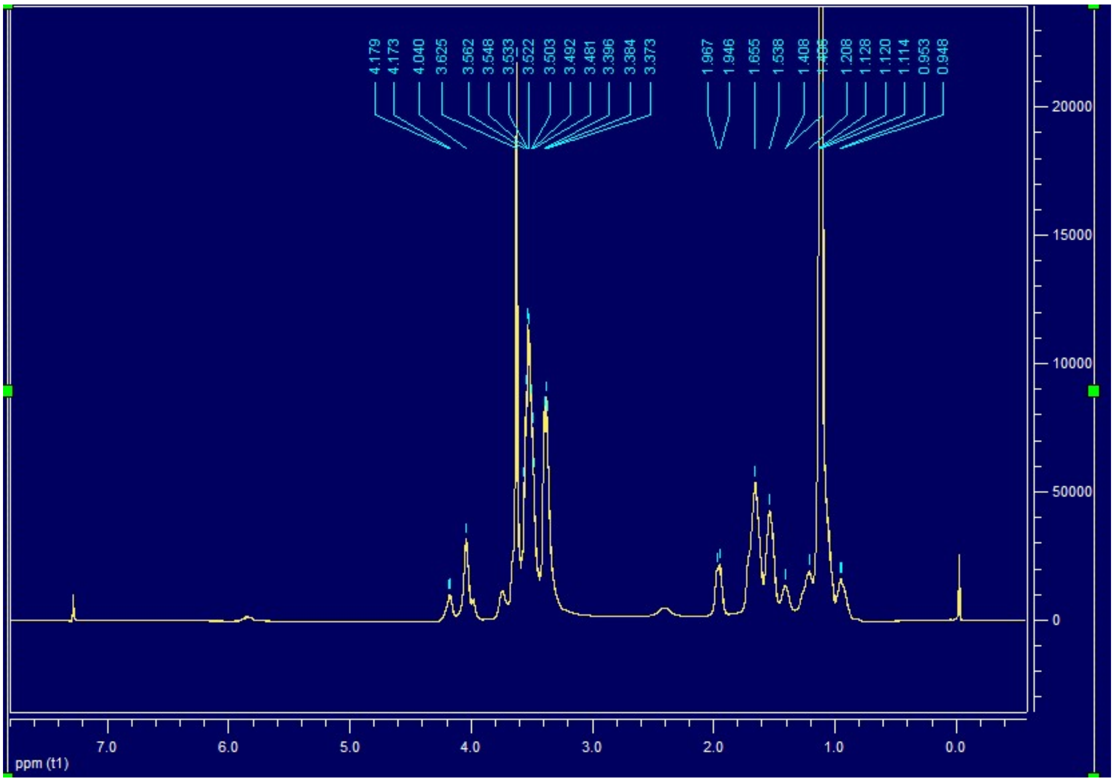

^{1}H NMR spectrum of PFPU0

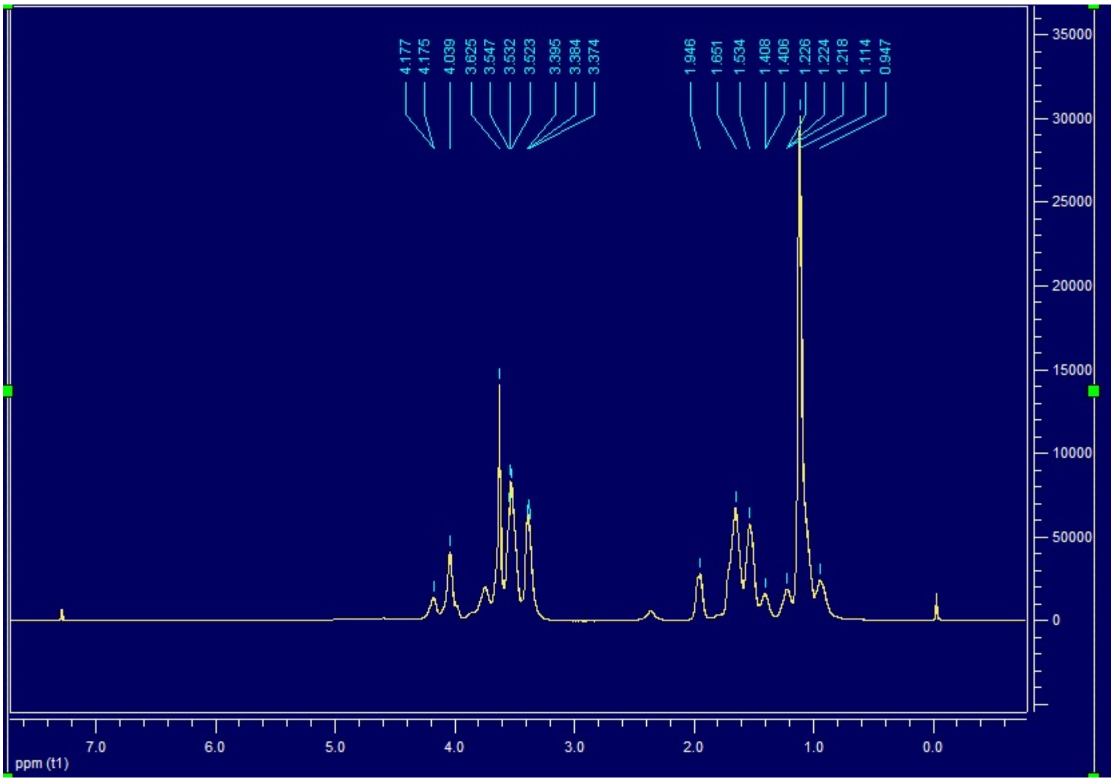

^{1}H NMR spectrum of PFPU8

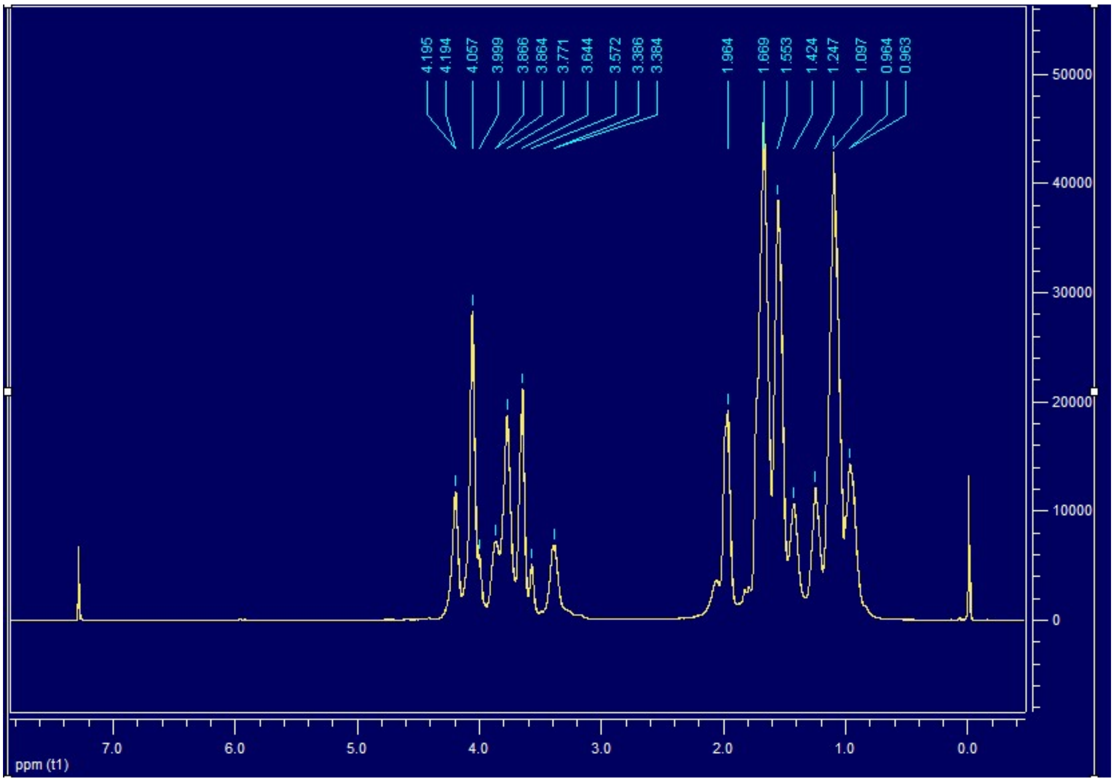

^{1}H NMR spectrum of PFPU20
